# Supplementary material for: Patient satisfaction with advanced physiotherapy practice internationally: Protocol for a systematic mixed studies review
Source: PLoS One. 2023 Oct 20;18(10):e0293170. doi: 10.1371/journal.pone.0293170 (PMC10588830; doi:10.1371/journal.pone.0293170)
Supplement: S1 Table — (DOCX) [file pone.0293170.s001.docx]

**S1 table: Adapted search strategies**

**Caption: S1 table. Adapted search strategies.**

**Embase**

| 1 | patient satisfaction/ |
| --- | --- |
| 2 | ((patient* or client* or participant*) adj5 (experience* or perception* or perceive* or expectation* or happiness or contented* or fulfilment)).tw,kf. |
| 3 | Satisf*.tw,kf |
| 4 | 1 or 2 or 3 |
| 5 | physiotherapist/ |
| 6 | physiotherapy/ |
| 7 | Physiotherapy practice/ |
| 8 | physiotherap*.tw,kf. |
| 9 | physio-therap*.tw,kf. |
| 10 | physical therap*.tw,kf. |
| 11 | 4 or 5 or 6 or 7 or 8 |
| 12 | 4 and 11 |
| 13 | advanced practice provider/ |
| 14 | ((advanced or speciali* or consultant* or extended or expanded) adj5 (practice or practitioner* or scope or role or physi*)).tw,kf. |
| 15 | (First adj3 contact adj3 (physiotherap* or physio-therap* practitioner*)).tw,kf |
| 16 | military physician extender*.tw,kf. |
| 17 | senior physiotherapist fellow*.tw,kf. |
| 18 | 13 or 14 or 15 or 16 or 17 |
| 19 | 12 and 18 |

**CINAHL**

| S18 | S12 AND S17 |
| --- | --- |
| S17 | S13 OR S14 OR S15 OR S16 |
| S16 | (First n3 contact n3 (physiotherap* or physio-therap* practitioner*)) |
| S15 | “senior physiotherapist fellow” |
| S14 | military physician extender* |
| S13 | ((advanced or speciali* or consultant* or extended or expanded) n5 (practice or practitioner* or scope or role or physi*)) |
| S12 | S4 AND S11 |
| S11 | S5 OR S6 OR S7 OR S8 OR S9 OR S10 |
| S10 | physical therap* |
| S9 | physio-therap* |
| S8 | Physiotherap* |
| S7 | (MH "Physical Therapy Practice") |
| S6 | (MH "Physical Therapy") |
| S5 | (MH "Physical Therapists") |
| S4 | S1 OR S2 OR S3 |
| S3 | Satisf* |
| S2 | ((patient* or client* or participant*) n5 (experience* or perception* or perceive* or expectation* or happiness or contented* or fulfilment)). |
| S1 | (MH "Patient Satisfaction") |

**Web of Science**

| 15 | #14 AND #9 |
| --- | --- |
| 14 | #10 OR #11 OR #12 OR #13 |
| 13 | TS=(senior physiotherapist fellow*) |
| 12 | TS=(military physician extender*) |
| 11 | TS=(First AND contact) near/6 (physiotherap* or physio-therap* practitioner*)) |
| 10 | TS=((advanced or speciali* or consultant* or extended or expanded) near/5 (practice or practitioner* or scope or role or physi*)) |
| 9 | #8 AND #4 |
| 8 | #7 OR #6 OR #5 |
| 7 | TS=(physio-therap*) |
| 6 | TS=(Physiotherap*) |
| 5 | TS=(Physical therap*) |
| 4 | #1 OR #2 OR #3 |
| 3 | TS=(satisf*) |
| 2 | TS=((patient* or client* or participant*) near/5 (experience* or perception* or perceive* or expectation* or happiness or contented* or fulfilment)) |
| 1 | TS =(patient satisfaction) |

**Cochrane**

| #1 | MeSH descriptor: [Patient Satisfaction] explode all trees |
| --- | --- |
| #2 | ((patient* or client* or participant*) near/5 (experience* or perception* or perceive* or expectation* or happiness or contented* or fulfilment)):ti,ab,kw |
| #3 | Satisf* |
| #4 | 1 or 2 or 3 |
| #5 | MeSH descriptor: [Physical Therapists] explode all trees |
| #6 | MeSH descriptor: [Physical Therapy Modalities] explode all trees |
| #7 | MeSH descriptor: [Physical Therapy Specialty] explode all trees |
| #8 | Physiotherap*:ti,ab,kw |
| #9 | physio-therap*:ti,ab,kw |
| #10 | Physical therap*:ti,ab,kw |
| #11 | 5 or 6 or 7 or 8 or 9 or 10 |
| #12 | 4 and 11 |
| #13 | ((advanced or speciali* or consultant* or extended or expanded) near/5 (practice or practitioner* or scope or role or physi*)):ti,ab,kw |
| #14 | (First near/3 contact near/3 (physiotherap* or physio-therap* practitioner*)):ti,ab,kw |
| #15 | military physician extender*:ti,ab,kw |
| #16 | senior physiotherapist fellow*:ti,ab,kw |
| #17 | 13 or 14 or 15 or 16 |
| #18 | 12 and 17 |
