# Supplementary material for: Patient satisfaction with advanced physiotherapy practice internationally: Protocol for a systematic mixed studies review
Source: PLoS One. 2023 Oct 20;18(10):e0293170. doi: 10.1371/journal.pone.0293170 (PMC10588830; doi:10.1371/journal.pone.0293170)
Supplement: Attachment — Submitted filename: APP PSAT SMSR rebuttal letter PLOS ONE Sept 2023.docx [file pone.0293170.s003.docx]

School of Graduate and Postdoctoral Studies,

Faculty of Health Sciences,

Western University,

London, Ontario,

Canada.

Date: Sept 26^th^, 2023.

Dear Mr. Maher Abdelraheim Titi, Mr. Simon Lafrance, and other reviewers;

We were pleased to receive your email on September 4^th^ 2023 inviting us to resubmit our manuscript (PONE-D-23-21887) titled “Patient satisfaction with advanced physiotherapy practice internationally: protocol for a systematic mixed studies review” following suggested revisions.

I would like to take this opportunity to thank you for your thorough and valuable feedback, which will undoubtedly improve the quality of our manuscript. The revisions are saved as tracked changes in the uploaded manuscript file and are explained after the word “**Response:**” in the point-by-point responses below. A clean file of the manuscript is also uploaded.

Responses to the editor

1. “Please ensure that your manuscript meets PLOS ONE's style requirements, including those for file naming”.

**Response**: We thank the editor for this feedback and have changed the naming of supplementary files to ensure our manuscript is compliant with style requirements.

2. “In your Data Availability statement, you have not specified where the minimal data set underlying the results described in your manuscript can be found”.

**Response**: As a protocol, it was an oversight for us to mark the data reporting section as not applicable. We have now included a data reporting and availability statement (line 480) for clarity.

3. “Please review your reference list to ensure that it is complete and correct”.

**Response**: Following revisions, and based on reviewer feedback, we have revised our reference list to ensure accuracy and completeness.

Responses to reviewer 1

1. #INTRODUCTION *Background: The authors analyse the concepts of patient satisfaction and patient experience in the context of physiotherapy. Although the work they do is commendable, I believe it is appropriate to integrate some fundamental references for both concepts into the background. Among them, I suggest: patient satisfaction (doi: 10.1080/09638288.2018.1501102), patient experience (doi: 10.1093/ptj/pzac080 doi: 10.1186/s40945-020-00088-6).

**Response**: Thank you for bringing these important papers to our attention. The patient satisfaction paper adds value and strengthens the introduction to our paper. We have therefore included a new paragraph (lines 69-72) and referenced within. The patient experience paper focuses more on lived experience of people with neck pain, rather than the process indicator of quality and we have therefore not included it.

2. #METHODS *Tidier: Please report it in full.

**Response**: We agree this adds to the completeness of the Tidier checklist and have included boxes (line 115) for Modifications, How well (planned), and How well (actual).

3. #METHODS *Search strategy: Please report the search strategy for each database.

**Response**: Although not required by PRISMA, we agree this improves transparency and have included additional search strategies as supplementary file 1 (S1 table).

4. #METHODS *Research group details: Please report details about the authors' background. Are they pt? Have they experienced in performing mixed-method systematic reviews?

**Response**: Given the qualitative component to our systematic mixed studies review, we agree that researcher positionality is integral to a robust methodology. We have therefore added a researcher positionality statement (lines 160-167) to provide pertinent information about the author group to the reader.

5. #FIGURE *Figure 1: Please report in full the abbreviation TBC.

**Response**: This was an oversight on our part and appreciate it may not be clear to all readers. To remedy this, we have reported the full abbreviation after the PRISMA flow diagram (line 225).

Responses to reviewer 2

1. Major comment: In your eligibility criteria you include “APP (…) have post-registration masters level specialization”, however, many studies include APP without this specialization and who instead has a bachelor level education with an onsite APP training such as a residency type training. Other studies poorly described the training of APP, sometimes because many APP with different training are included. Therefore, this inclusion criterion, if taken by its words, would lead to significant number of relevant papers to be excluded from your analysis. It is also important to make the distinction between advanced practice physiotherapy roles, which does not necessarily require a specific training or highly specialized training and advanced practice physiotherapy title, that are for example not present in Canada.

**Response**: Thank you for this important comment. We agree the original manuscript did not make this clear and have therefore made the following edits. We amended the order and added a paragraph into the rationale (lines 95-98) to reflect that variation exists internationally regarding educational requirements. We have also added “or equivalent” to follow masters level education/specialisation throughout (lines 41, 91, 117, 189) to reinforce this important message.

2. Minor comments / suggestions: Lines 29-30 “However, no evidence synthesis of patient satisfaction with APP across clinical fields exists.” This is no completely true as a few systematic review included a synthesis of patient satisfaction, at least of quantitative questionnaires. You also discussed results from a few studies in the introduction.

**Response**: We agree this wording could be unclear and did not emphasise the “across clinical fields” message enough. We have therefore re-written this sentence (lines 29-30) to place more onus on “clinical fields” and improve clarity.

3. Lines 39-40 & 85-86 “b) have post-registration masters level specialisation” Does that mean that you will exclude all studies including advanced practice physiotherapists that do not have a masters level specialisation? If so, you will exclude some relevant studies as many include physiotherapists with a bachelor level education who received a non-master-level APP training (e.g., residency type training). Please consider removing this eligibility criteria or clarifying the text.

**Response**: We agree this was not clear and hope our changes from Major Comment 1 have addressed this.

4. Lines 44-45: Please provide more details regarding the data synthesis plan.

**Response**: Although we agree the detail pertaining to synthesis is limited at this stage of the manuscript, we aren’t able to make any edits due to the abstract word limit.

5. Line 103 – Table 1: Although they were reported in the Tawiah et al. paper, are ‘Manual Therapists’, ‘Military Physician Extender’ and ‘Senior Physiotherapist Fellow’ really terms used to describe APP? I would suggest either to remove them from Table 1 or to provide a footnote providing more details (e.g., these terms have been used in the past to describe APP although …).

**Response**: We agree these are terms are rarely used to describe APP and feel your suggestion of a footnote is excellent. We have included this (lines 114-115) and based it on content from the original Tawiah et al paper that stated these only accounted for 28% of titles used internationally.

6. Line 105 – Table 2: in the What (procedures), we see treatment and rehabilitation that are under the scope of PT and may or may not be included in an APP model of care (although they are often included). Should a distinction be made between APP procedures (like the other ones that are described) and PT procedures that may be included in an APP model of care?

**Response**: We have used a footnote (line 119) to separate/highlight PT procedures that may be included in APP. We based this on World Physiotherapy’s description of PT.

7. Line 105 – Table 2: As mentioned above, it is not necessary to be an “highly experienced, physical therapists with post-registration master’s level (or higher)” to be considered as an advanced practice physiotherapist or at least to work in an advanced practice role.

**Response**: Agreed, and we hope this has been addressed above.

8. Lines 112-113: You discussed well the results from previous systematic reviews. However, there is two limitations in the previous systematic review that I think you should highlight. First, none of them conducted a comprehensive evaluation of patient satisfaction with APP care, instead they all focused on various outcomes specific to a clinical setting (specialized medical care or emergency department) which limit the evaluation of satisfaction to these settings. Also, they only included quantitative data, omitting to include qualitative analysis from patients interviews, for example. I think you can also cut a bit the description of these studies, especially if you are lacking word space.

**Response**: These are two important limitations and help build a clear justification for our review highlighting the gap in the existing literature. To address these points, we have made edits to the introduction (lines 121-122, 148-149, and 151).

9. Line 159 – Table 3: Intervention same comment as above regarding the masters level specialisation

**Response**: Agreed, and we hope this has been addressed above.

10. Line 159 – Table 3: Study design, you could add a few examples e.g., such as experimental, observational or qualitative studies

**Response**: We agree this will add clarity to the reader so have included three examples within Study Design (line 189).

11. Line 159 – Table 3: Which software? Also in the final article, it would be important to provide details regarding the studies that were translated and included or excluded because translation was not possible.

**Response**: Thank you for bringing this oversight to our attention. We have now added Google Translate as our software of choice. In the review manuscript we will include the details you highlight.

12. Lines 172-175: You provide the search strategy for MEDLINE and mentioned that the search strategy was adapted for other included databases. Although it is not of common practice, it would be even more transparent and useful to other researchers who might want to replicate your study to provide all search strategies in supplementary materials (at least for the paper publication).

**Response**: Although not required by PRISMA, we agree this improves transparency and have included additional search strategies as supplementary file 1 (S1 table).

13. Lines 280-282: You mention that a “constructivist paradigmatic approach” will be used. Although you described why you would use it, I believe that readers would benefit from having a short description of what is a “constructivist paradigmatic approach”. Also, the reference 40 (Thomas & Harden 2008) does not describe this approach.

**Response**: Thank you for this suggestion, we agree the readers may benefit from a clearer description of this paradigm, especially given some of the complexities surrounding research paradigms. We have included an extra paragraph to address this (lines 313-315). To your point regarding referencing, we have also amended and feel our references now clearly support their corresponding statements.

We believe all the changes made to our manuscript have improved its overall quality and would like to thank you once again for your time and thoroughness with this review. We hope our changes and comments are satisfactory and our manuscript can now progress towards publication.

If you have any further questions, or wish to discuss anything in more depth, please don’t hesitate to get back in contact via [cdavi2@uwo.ca](mailto:cdavi2@uwo.ca).

Kind Regards,

**Chris Davis**

MSc, BSc (Hons), MCSP, HCPC.

PhD student, Health and Rehabilitation Sciences.
